# A Cross-Sectional Study of Facilitators and Barriers of Iranian Nurses’ Participation in Continuing Education Programs

**DOI:** 10.5539/gjhs.v6n2p183

**Published:** 2013-12-27

**Authors:** Zeinab Hamzehgardeshi, Zohreh Shahhosseini

**Affiliations:** 1 Department of Reproductive Health, Nasibeh Nursing and Midwifery Faculty, Mazandaran University of Medical Sciences, Sari, Iran

**Keywords:** barriers, continuing education, facilitators, nurses, Iran

## Abstract

**Background:**

Continuing education is one of the modern strategies to maintain and elevate knowledge and professional skills of nurses which in turn elevate the health status of society. Since several factors affect nurses’ participation in continuing education, it’s essential to know promoters and obstacles in this issue and plan accordingly.

**Methods:**

In this cross-sectional study, 361 Iranian nurses who were recruited by convenience sampling method completed an anonymous, self-administered questionnaire from October 2012 to April 2013. Topics covered the participants’ attitudes towards facilitators and barriers of their participation in continuing education.

**Results:**

Mean and standard deviation of participants’ age were 37.14±7.58 years and 93.94% were female. The maximum score of facilitators and barriers to nurses’ participation in continuing education were related to “Update my knowledge” and “Work commitments” respectively. The results showed among Iranian nurses, the mean score of personal and structural barriers was significantly higher than the mean score of interpersonal ones (F=2122.66, p<0.001).

**Conclusion:**

Results highlight policy makers and nursing managers’ role on improving the accessibility to provided continuing education programs by enforcement of facilitators and reducing barriers focusing on the personal and structural barriers.

## 1. Introduction

Human resources are the most important inputs to the healthcare system, and performance of healthcare system ultimately depends on knowledge, skill, and motivation of people responsible for providing these services (Richards & Potgieter, 2010). Accordingly, continuing education (CE) is the key tool to improve old and obsolete skills and to apply new technologies in providing services. Factors such as unprecedented growth in professional knowledge, desire for career promotion, development of nursing experiences, patients’ demand to receive services compatible with new standards, rapid changes in healthcare system, and consequently changes in nurses’ role, all emphasize the need for nurses to adjust to required professional changes and the necessity for designing CE programs in order to meet these learning requirements (Griscti & Jacono, 2006; Phillips, Piza, & Ingham, 2012). CE programs help nurses to improve their performance and keep pace with rapid changes (Patelarou, Vardavas, Ntzilepi, & Sourtzi, 2009). Although, due to lack of sufficient incentives for nurses participation in these courses and weakness in applying learnt subjects in the workplace, these programs may intensify disagreements and conflicts between nurses and their managers and policy makers (Ebrahimi et al., 2012).

On the other hand, CE programs are effective when they are based on staff’s real needs and they participate in CE voluntarily. In addition, needs assessment in this issue as well as assessment of participants’ expectations are important factors in determining educational objectives and implementation of CE programs (Vaezi, Vanaki, & Ahmadi, 2013). Accordingly, assessment of nurses’ views in relation to facilitators and barriers to their participation in these programs can largely help to meet these needs and expectations, and to improve the quality of these programs.

Various studies reveal that nurses take part in CE programs for many reasons including: promotion of professional knowledge and clinical skills, providing better care services for patients, up-dating their information, fulfilling organizational commitments, and obtaining educational certificates (Chong, Sellick, Francis, & Abdullah, 2011; Ebrahimi et al., 2012; Ni et al., 2013; Nsemo, John, Etifit, Mgbekem, & Oyira, 2013). Also, barriers to nurses’ participation in CE programs include lack of nursing manager’s and co-workers’ support, time constraint, unpleasant experiences of previous programs, and cost of courses (Chong, Sellick, Francis, & Abdullah, 2011; Ni et al., 2013; Penz et al., 2007; Schweitzer & Krassa, 2010).

Currently, participation in CE programs on the job is compulsory for nurses in many countries including Iran (Ebrahimi et al., 2012; Flores Peña & Alonso Castillo, 2006). Despite the long history of CE programs in Iran, conducted studies indicate relative dissatisfaction of nurses with these programs (Ebadi, Vanaki, Nehrir, & Hekmatpou, 2007; Ebrahimi et al., 2012). Because the studies show that compulsory CE compared to voluntary programs are less effective in elevating participants’ motivation for participation in these programs (Ebadi et al., 2012; James & Francis, 2011), and that many factors affect participation of nurses in CE programs, this study was established with the aim of assessment of facilitators and barriers to nurses’ participation in CE programs.

## 2. Methods

### 2.1 Setting and Data Collection

This cross-sectional descriptive study was conducted in Sari city, Mazandaran province, in the North of Iran. Sampling was recruited by convenience sampling method from those nurses who attended CE programs which were provided as a routine scheduled project during six months periods from October 2012 to April 2013. From 398 questionnaires which were distributed, data of 37 questionnaires were not entered to SPSS as a result of large missing data (response rate: 90.70%). Finally data of 361 nurses were reported.

#### 2.1.1 Instrument

The research instrument was developed by researchers based on a review of previous studies in this area (Chong et al., 2011; Ebrahimi et al., 2012; Ni et al., 2013) and the point of view of experts. The questionnaire used consisted of two parts. The first part was related to socio demographic information of participants (6 questions) and the second part included some questions about facilitators (15 questions) and barriers (19 questions in three domains of personal, interpersonal and structural factors) around nurses’ participation in CE programs in five point Likert scale. They completed this anonymous, self-administeredquestionnaire 10 minutes before their participation in scheduled CE programs.

The questionnaire psychometric properties were determined by content validity and reliability. In this way, Scale-Level content validity index of 0.90 for the instrument was obtained with the assistance of 10 faculty members of a medical sciences university, in the area of nursing, continuous education and instrument development through face-to-face semi-structured interviews. Reliability of instrument was demonstrated with Cronbach’s alpha coefficient = 0.92 in a sample of 20 nurses which were selected by convenience sampling method. Also, consistency of instrument was established with test-retest reliability with interval of 2 weeks (Intra Cluster Correlation= 0.93, P<0.001).

#### 2.1.2 Sample Size

In order to calculate the sample size, a mean prevalence of 64% for Iranian nurses’ participation in CE programs was considered, as shown in previous studies (Ebrahimi et al., 2012). With this prevalence, bearing the statistical error = 0.05 (which is an acceptable error), and the precise = 0.005, the sample size would then have to be at least 360.

### 2.2 Data Analysis

The collected data were fed into Statistical Package for Social Sciences for Windows version13.0 (SPSS Inc., Chicago, IL, USA) for further analysis. Descriptive statistics were reported as relative and absolute frequency as well as mean and standard deviation. Moreover, in order to compare the mean score of facilitator and barrier factors, an independent t-test, and a one-way analysis of variance were performed. In this case, p<0.05 was considered significant.

## 3. Results

Of the whole sample (361 cases), 93.94 % of them were female, and the rest were male. The mean and standard deviation of participants’ age and their employment record were 37.14±7.58 and 11.48±4.47 years respectively. Socio-demographic characteristics of the nurses are presented in Table 1.

**Table 1:** Demographic characteristics of the participants (N=361)

Age (yr)*		37.14 ± 7.58
Employment record (yr)*		11.48±4.47
Educational level**	Two trained courses after diploma	8(2.23)
	Baccalaureate	311(86.11)
	Masters/doctorate	42(11.66)
Marital status **	Married	72(19.91)
	Single	279(77.21)
	Divorced/Widow	10(2.88)
Gender	Female	339(93.94)
	Male	22(6.06)
Current work setting **	General hospital	232(64.35)
	Primary health care center	109(30.21)
	Private hospital	20(5.54)

* Mean±SD;

** Frequency (percent).

Based on the findings of this study, the mean score of facilitators to nurses’ participation in CE was significantly higher than the mean score of barriers (61.99±10.85 versus 51.17±12.83; p<0.001, t=12.23). The results of this research showed that the highest mean score of facilitators of nurses’ participation in CE was related to “Update my knowledge” (Table 2). Also the highest mean score of barriers according to participants’ point of view, in three personal, interpersonal and structural domains, were related to “Time constraints”, “ Lack of co-workers’ support” and “ work commitments”, accordingly (Table 3).

**Table 2:** Mean score and standard deviation of facilitators to nurses’ participation in CE

Facilitators	Mean	Standard Deviation
Update my knowledge	4.48	0.81
Give qualified care to patients	4.39	0.85
Improve my skill in clinical practice	4.35	0.90
Obtain knowledge to achieve personal status	4.34	0.81
Improve my teaching skills	4.31	0.90
Increase my competency	4.22	0.91
Improve my decision making skills	4.14	1.01
Improve my communication skills	4.13	0.99
Prevent myself from getting bored with routine	4.10	0.96
Boost my self-esteem	4.04	1.15
Improve my research skills	3.99	1.08
Gain more paper qualification	3.91	1.07
Improve my management skills	3.89	1.04
Adhere to hospital policy	3.86	1.04
Be more critical in providing nursing care	3.77	1.11

**Table 3:** Mean score and standard deviation of barriers to nurses’ participation in CE

Barriers		Mean	Standard Deviation
**Personal barriers**	Time constraint	3.24	1.18
	Domestic responsibilities	2.86	1.18
	Emotional stress	2.12	1.16
	Poor physical health	1.91	1.10
**Interpersonal barriers**	Lack of co-workers’ support	2.74	1.17
	Negative experiences with previous CE programs	2.29	1.15
	Lack of family support	2.23	1.20
	Poor interaction of CE programs’ staff	1.91	1.12
**Structural barriers**	Work commitments	3.50	1.24
	Cost of courses	3.32	1.17
	Geographic distance	3.03	1.25
	Poor scheduling of CE programs	3.00	1.15
	Lack of organizational support	2.95	1.29
	Lack of information about provided CE programs	2.93	1.18
	Lack of accessibility to provided CE programs	2.91	1.12
	Lack of supervisors’ support	2.89	1.23
	Lack of relevant CE programs	2.82	1.07
	Poor quality of provided CE programs	2.49	0.99
	Needs satisfied by on the job training	1.95	1.09

In order to answer the question “what is the most priority domain of barriers to participation in CE among Iranian nurses?” A one-way analysis of variance was conducted. The results showed the mean score of personal and structural barriers was significantly higher than the mean score of interpersonal ones (F=2122.66, p<0.001) (Table 4).

**Table 4:** Analysis of variance between barriers to nurses’ participation in CE

Barriers	N	Mean± SD	95% Confidence Interval for Mean		F	Sig
			Lower Bound	Upper Bound		
**Personal**	361	10.15±3.36	9.80	10.50	2122.66	p<0.001
**Interpersonal**	361	9.18±3.37	8.83	9.53		
**Structural**	361	31.83±7.80	31.02	32.64		
**Total**	1081	17.05±11.71	16.35	17.75		

## 4. Discussion

CE programs are one of the professional principles in health-related disciplines, including nursing. To increase nurses’ skills and achieve their professional goals, they should take part in CE programs. With the possibility to assess nurses’ perspectives on facilitator and barriers to their participation in CE programs, and thus, plan accordingly to create strong incentives for their active participation (not merely as organizational requirement), would help provide better care for patients. Still, it seems the two phenomena of facilitator and barriers factors can have mutual effects on each other, and perhaps the best way to investigate them, is to study them together. Thus, this study was conducted to investigate these factors from the point of Iranian nurses.

Results obtained in this study indicated higher mean score of facilitator to participate in CE programs compared to barriers which emphasized that for programs to succeed, reinforcing incentives should be more considered by nursing managers and policy makers than reducing obstacles. Also, findings of this study in relation to “Update my knowledge” as the highest facilitator for participation in CE programs in the opinion of Iranian nurses are in line with studies that show nurses like to take part in programs with more learning and applicability (Chong et al., 2011; Griscti & Jacono, 2006; Ni et al., 2013). As one of the most important factors in improvement of nurses, CE programs prepare them for progress and efficiency in present and future professional positions during service, and modify their way of thinking and performing, and give them access to relevant information based on needs to achieve organizational objectives (Richards & Potgieter, 2010).

Participating nurses in this study considered organizational barriers as the most barriers for their participation in CE programs, and among these barriers, nurses’ commitment to their workplace had the highest mean score. This finding, along with barriers such as time constraint, and lack of co-workers’ support (in relation to swapping work shift in order to create opportunity for attending education programs); although in line with results of other studies in this area (Aiga, 2006; Ni et al., 2013), Iranian nurses may still have to allocate little time to CE due to pressing workload and shortage of personnel (Farsi, Dehghan Nayeri, Negarandeh, & Broomand, 2010). Financial difficulties and subsequent compulsory overtime diminishes nurses’ energy and leaves no time or opportunity to participate in CE programs (Ebadi et al., 2007). Thus, it is recommended that more coordination between policy makers in the area of CE programs and nursing managers should take place in order to regulate working shifts.

In conclusion given the key role played by nurses in the healthcare system, and that this role can be played well with professionally qualified nurses, and to maintain, improve, and continue such qualification, continuing efficient education is required. Explaining strategies used in CE programs for nurses based on facilitating incentives and reducing obstacles can help managers, planners, and executors of CE to design and implement effective CE, and provide easier achievement of CE objectives which are to improve and enhance performance of nurses.

Limitations in this study included personal differences and participants’ sincerity in answering questions, which can affect the results of the study. Another limitation in this study was that facilitator and barriers to participation in CE programs were only investigated from the perspective of nurses. In future studies, it is recommended that further comparative studies, based on opinions of nursing managers and policy makers and executors of CE programs be conducted. Despite these limitations, since participating nurses in this study were from public and private hospitals and health centers, results obtained can add to the depth of our previous knowledge in relation to factors affecting participation of nurses in CE programs.
